# A Case of Atypical Lipomatous Tumor that Dedifferentiated with Second Recurrence after Additional Resection

**DOI:** 10.7759/cureus.2954

**Published:** 2018-07-10

**Authors:** Kazuhiko Hashimoto, Shunji Nishimura, Masao Akagi

**Affiliations:** 1 Orthopedic Surgery, Kindai University Hospital, Osakasayama, JPN; 2 Orhtopedic Surgery, Kindai University Hospital, Osakasayama, JPN

**Keywords:** liposarcoma, extended resection, mesenchymal tumor, atypical lipomatous tumor

## Abstract

Dedifferentiated liposarcoma is recognized as a type of liposarcoma that usually occurs concomitantly with the well-differentiated type. In this report, we discuss the case of a 65-year-old man who developed a dedifferentiated liposarcoma with second recurrence of an atypical lipomatous tumor. The patient first presented to us with an atypical lipomatous tumor of the right elbow for which he underwent a marginal resection. After five months, the patient experienced tumor recurrence for which he underwent another extended resection. Approximately 10 months following this surgery, the tumor recurred a second time for which he underwent another extended resection. Histopathological analysis of the second recurring tumor revealed a dedifferentiated liposarcoma. So far, two years following this resection, recurrence has not been observed. This is the first case of an atypical lipomatous tumor that dedifferentiated after the additional extended resection.

## Introduction

A liposarcoma is a malignant mesenchymal tumor that can present as an atypical lipomatous tumor, well-differentiated liposarcoma, dedifferentiated liposarcoma, myxoid liposarcoma, and pleomorphic liposarcoma [1]. In 1979, Evans suggested that a dedifferentiated liposarcoma was similar to a differentiated adipose sarcoma [2], which frequently occurs in limbs and the retroperitoneum and is more likely to affect patients aged 40 to 60 years [1, 3]. The dedifferentiated tumor is generally well understood but it is very rare; it occurs in less than 5% of well-differentiated liposarcomas and rare tissue types [4]. In addition, research shows that it usually occurs concomitantly with the well-differentiated type [5]. So far, no cases of atypical lipomatous tumor that dedifferentiated with recurrence after additional extended resection have been reported. We describe a very rare case of dedifferentiated liposarcoma that occurred in a patient following additional resection for an atypical lipomatous tumor.

## Case presentation

**Figure 1:**
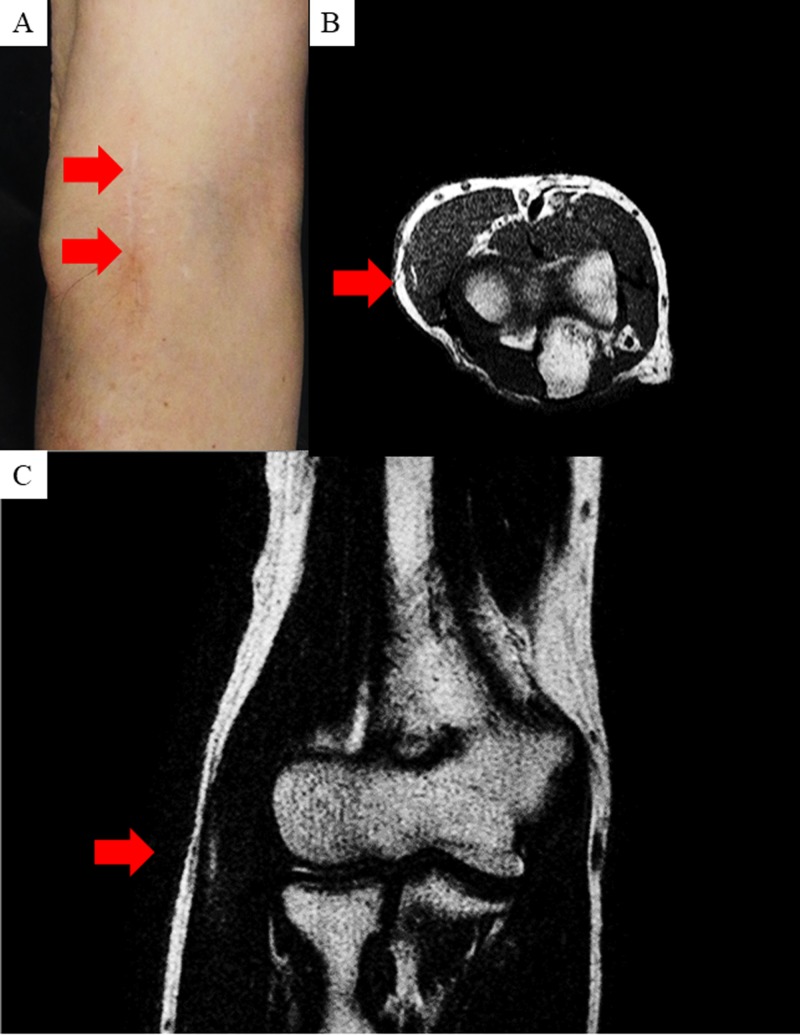
Appearance and magnetic resonance imaging (MRI) features of the right elbow. Appearance of the right elbow after the first resection (A). MRI shows the right elbow after the first resection with coronal (B) and sagittal sections (C). Small region of high intensity is observed and they favor some inflammation after the surgery (B and C).

A 63-year-old man presented with a 2 x 2 cm mass on the lateral side of his right elbow. Previously, he had visited a nearby doctor, and was then referred to us for a surgical consultation. After his surgical consultation, the patient underwent a marginal resection of the mass (Figure 1A). The histological analysis revealed an atypical lipomatous tumor (data not shown). After the resection, the patient was assessed with magnetic resonance imaging (MRI) which showed no remains of the tumor (Figure 1B, 1C).

**Figure 2:**
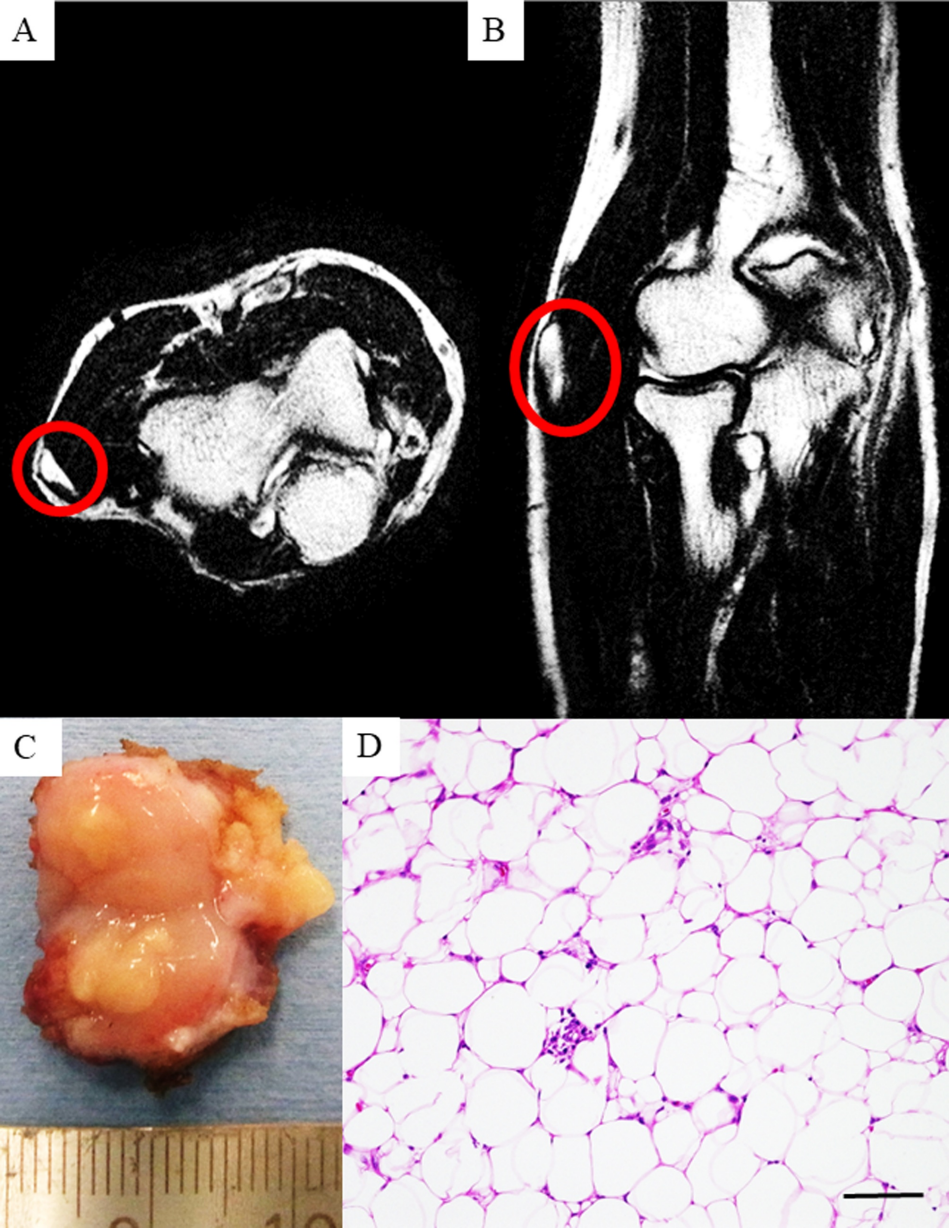
Feature of the recurrence tumor. Magnetic resonance imaging (MRI) shows the right elbow after recurrence with sagittal (A) and coronal sections (B). The resected specimen after recurrence (C). Histology of the resected specimen after recurrence (D). Scale bar = 100 μm.

Approximately 10 months following this resection, tumor recurrence was identified on MRI (Figure 2A, 2B). He was then referred to our hospital for a surgical consultation. We performed a wide resection. The patient had received no targeted therapy, chemotherapy prior to resection or after.

The resected specimen was yellow-white in color and had a hard, elastic texture (Figure 2C). A histological analysis of the resected specimen revealed an atypical lipomatous tumor (Figure 2D), and the tumor margin was negative.

**Figure 3:**
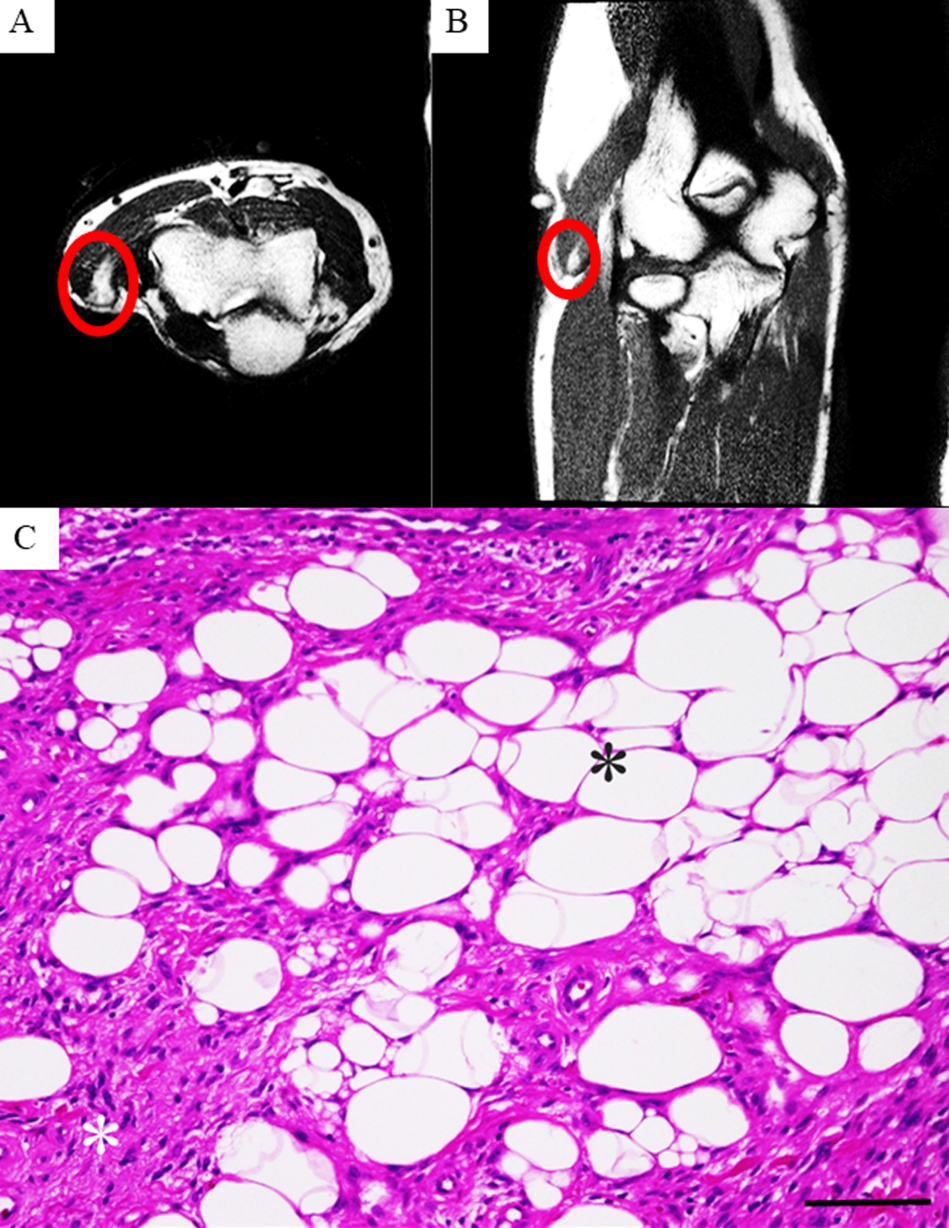
Features of the second recurrence tumor. Magnetic resonance imaging (MRI) features of the right elbow after the second recurrence with sagittal (A) and coronal sections (B). Histology of the resected specimen (C). Atypical lipomatous cells (black asterisk). High grade spindle cells (white asterisk). Scale bar = 100 μm.

One year following this surgery, the tumor recurred as indicated on MRI imaging (Figure 3A, 3B). Again, we treated the patient by performing an extended resection. This time, the histological analysis revealed a proliferation of atypical lipomatous cells and high-grade spindle cells (Figure 3C).

We diagnosed dedifferentiated liposarcoma based on the histological findings. So far, two years have passed, and no recurrence has been observed.

## Discussion

Dedifferentiated liposarcoma usually coexists with well-differentiated liposarcoma or recurs after resection of well-differentiated liposarcoma [5, 6]. An earlier report discussed how a dedifferentiated liposarcoma occurred after performing two marginal resections in the patient [7]. However, no reports have described a case of dedifferentiated liposarcoma after extended resection.

Dedifferentiated tumors generally indicate some differentiated traits; however, cells and tissues eventually lose their differentiation traits and develop into an undifferentiated state, which enhances the proliferative capacity of the tissue. In general, when a liposarcoma dedifferentiates, its histology shows the features of malignant fibrous histiocytoma (MFH). Research also suggests that the time to dedifferentiation is 7.7 years on average (1–23 years) [6]. In the current case, dedifferentiation occurred within 15 months of the initial resection. This short interval between the initial diagnosis and dedifferentiation is unusual. Interestingly, recent reports show that when a liposarcoma dedifferentiates rapidly, its histology shows a mosaic-pattern [6]. For example, dedifferentiated liposarcomas sometimes include components of an osteosarcoma, a leiomyosarcoma, and a chondrosarcoma. Histological features of the current case also showed combined well-differentiated and dedifferentiated components. The best approach for treating dedifferentiated liposarcoma is extensive resection [8]. However, there are reports that demonstrate effective treatment approaches that use combined radiation therapy and surgery [9, 10]. No effective chemotherapy has been established. In general, the prognosis of dedifferentiated liposarcoma is poorer than that of liposarcomas with other histological features. Henricks et al. reported that the local recurrence rate, the metastatic rate, and the five-year survival rate for dedifferentiated liposarcoma are approximately 41%, 17%, and 28%, respectively [6]. Careful follow-up observation is necessary in the future. We have some limitations in the current study. First, we could not deny the possibility of remained tumor after additional resection in the MRI. We discussed with our radiologist and concluded that the high-intensity region will have inflammation after the surgery. Second, we did not perform the immunohistochemistry or fluorescent in situ hybridization (FISH) to detect the amplification of MDM2 and CDK4. However, we could diagnose with the histological findings of hematoxylin and eosin (H&E) staining.

## Conclusions

We described a case of atypical lipomatous tumor that dedifferentiated after the first recurrence and after additional resections of the well-differentiated tumor. All musculoskeletal oncologists should keep in mind that atypical lipomatous tumor can dedifferentiate with recurrence after additional resection.
